# A Research Translation, Implementation and Impact Strategy for the Australian Healthy Environments and Lives (HEAL) Research Network

**DOI:** 10.3390/ijerph20146383

**Published:** 2023-07-18

**Authors:** Katrina Lyne, Carmel Williams, Sotiris Vardoulakis, Veronica Matthews, Brad Farrant, Andrew Butt, Iain Walker, Cordia Chu, Martine Dennekamp, Daniela A. Espinoza Oyarce, Rebecca Ivers, Bin Jalaludin, Penelope J. Jones, Karina Martin, Lucie Rychetnik

**Affiliations:** 1 Centre for Health in All Policies Research Translation, Health Translation SA, SAHMRI, Adelaide, SA 5000, Australia; 2 School of Public Health, University of Adelaide, Adelaide, SA 5000, Australia; 3 College of Health and Medicine, Australian National University, Canberra, ACT 2601, Australia; 4 University Centre for Rural Health, University of Sydney, Lismore, NSW 2480, Australia; 5 Telethon Kids Institute, University of Western Australia, Perth, WA 6000, Australia; 6 Centre for Urban Research, RMIT University, Melbourne, VIC 3000, Australia; 7 Melbourne Centre for Behaviour Change, University of Melbourne, Parkville, VIC 3010, Australia; 8 Centre for Environment and Population Health, School of Medicine and Dentistry, Griffith University, Southport, QLD 4222, Australia; 9 Environment Protection Authority Victoria, Carlton, VIC 3053, Australia; 10 School of Population Health, Faculty of Medicine and Health, University of New South Wales, Sydney, NSW 2052, Australia; 11 Menzies Institute for Medical Research, University of Tasmania, Hobart, TAS 7000, Australia; 12 The Australian Prevention Partnership Centre, Sax Institute, Haymarket, NSW 1240, Australia; 13 School of Public Health, University of Sydney, Sydney, NSW 2006, Australia

**Keywords:** research translation, knowledge exchange, environmental change, public health

## Abstract

Healthy Environments And Lives (HEAL) is the Australian national research network established to support improvements to health, the Australian health system, and the environment in response to the unfolding climate crisis. The HEAL Network comprises researchers, community members and organisations, policymakers, practitioners, service providers, and other stakeholders from diverse backgrounds and sectors. HEAL seeks to protect and improve public health, reduce health inequities and inequalities, and strengthen health system sustainability and resilience in the face of environmental and climate change, all with a commitment to building on the strengths, knowledge, wisdom, and experience of Aboriginal and Torres Strait Islander people, culture, and communities. Supporting applied research that can inform policy and practice, and effective research translation, implementation, and impact are important goals across the HEAL Network and essential to achieve its intended outcomes. To aid translation approaches, a research translation, implementation, and impact strategy for the HEAL Network was developed. The strategy has been created to inform and guide research translation across HEAL, emphasising communication, trust, partnerships, and co-design with communities and community organisations as well as the decision-makers responsible for public policies and programs. Development of the strategy was guided by research translation theory and practice and the Health in All Policies and Environment in All Policies frameworks. As described in this paper, the strategy is underpinned by a set of principles and outlines preliminary actions which will be further expanded over the course of the HEAL Network’s activities. Through these actions, the HEAL Network is well-positioned to ensure successful research translation and implementation across its program of work.

## 1. Introduction

The global climate crisis cannot be addressed by one state or sector in isolation; wider multi-sectoral collaboration is needed to identify examples of best practices in climate action and to explore and address challenges to their effective implementation [1]. Healthy Environments And Lives (HEAL) is the Australian national research network established to support improvements to health, the Australian health system, and the environment in response to the unfolding climate crisis [2]. Effective research translation and implementation are central to the objectives of the HEAL Network, which encompass improvements to public health, equity, and health system resilience. HEAL has a particular focus on building on the strengths, science, knowledge, and wisdom of Aboriginal and Torres Strait Islander people given their ancient and ongoing careful custodianship of Australian land and waters; as such, First Nations Knowledge Systems is a central theme. HEAL also has a strong focus on data science, policy, and community engagement purposes to develop the evidence base and decision support systems that will inform national and regional policies relevant to climate change and public health. This paper outlines HEAL’s approach to research translation, implementation, and impact to inform and support knowledge exchange across the network’s program of work.

## 2. Healthy Environments and Lives (HEAL Network)

The HEAL Network comprises researchers, community members and organisations, policy actors, practitioners, service providers, and other stakeholders from diverse backgrounds. It draws together a unique combination of existing research groups and collaborations in the fields of environmental and climate change and human health, leveraging expertise across various sectors. HEAL is funded by the National Health and Medical Research Council Special Initiative in Human Health and Environmental Change over five years from 2022 [3]. Similar international initiatives that bring together the climate and health sectors include the European project ENBEL (Enhancing Belmont Research Action to support EU policy-making on climate change and health) [4], the Lancet Countdown on health and climate change [5], and a range of World Health Organization initiatives aiming to build capacity and capability in relation to climate change and health [6].

The vision of HEAL is ‘to catalyse research, knowledge exchange and translation into policy and practice that will bring measurable improvements to our health, the Australian health system, and the environment’ [7]. HEAL aims to lead environmental change and health research to provide the evidence, capacity, capability, and tools urgently needed to achieve the following objectives:

- Protect and improve community health, especially at-risk groups and people in regions and communities disproportionately affected by environmental and climate change;
- Strengthen health system resilience, preparedness, and responsiveness to changing environmental conditions and related diseases, and reduce its environmental impact; and
- Reduce inequities and inequalities within and across communities and generations [7].

Examples of expected HEAL outcomes include the establishment of Communities of Practice in all Australian jurisdictions to provide fora for interaction, co-design, and knowledge exchange; completion of a national environmental health risk assessment that describes the burden of environmental and climate change on health in Australia; improved mapping of environmental changes and their impacts on communities by bringing together First Nations knowledge systems and Western science and data; and holistic evaluation of the effectiveness of targeted interventions which will support communities and policymakers to address environmental and health challenges.

Effective research translation and implementation are essential for the HEAL Network to achieve its objectives and outcomes. Across HEAL, a range of individuals and groups may contribute to and co-design research, translation, implementation, and impact, including but not limited to the following:

- Community organisations: independent, not-for-profit organisations that are initiated, based, governed, operated, and accountable to the community;
- Researchers: persons engaged in the creation of new research knowledge, for example, those working within universities or other institutions, as well as Aboriginal and Torres Strait Islander Elders and community researchers;
- Policy actors: persons involved in formulating or influencing policies, such as public sector employees, politicians, and persons working for non-government organisations;
- Practitioners: persons working primarily in policy implementation or service provision, for example, in health, environmental, or community services, and in government, non-government, or private sector organisations;
- Knowledge brokers and translators: persons actively working to support the translation of research into policy and practice, for example, in a boundary spanner or knowledge exchange role;
- Community members: interested members of the Australian community participating in the work of HEAL, but not in one of the roles listed above.

Given the diversity of contributors to the HEAL Network and the associated range of knowledge and experience in research translation, a subgroup was established in 2022 to guide and support research translation activities across HEAL. In its first iteration, the research translation subgroup comprises individuals from research, government, and non-government organisations representing a range of disciplines. A key objective of the subgroup is to support network members to engage with one another to co-produce useable research which facilitates timely policy and practice changes at local, regional, state, and national levels; capacity development is a central component. The work of the subgroup is informed by theory and practice across the paradigms of research translation.

## 3. Paradigms of Research Translation

For the HEAL Network’s purposes, research translation is described as the process by which knowledge and evidence derived from research are incorporated into policy, practice, and other decision-making processes. Many terms are used to describe the translation process that underpins evidence-informed policy, practice, and decision-making; these terms are often used interchangeably, for example, research translation, knowledge translation, knowledge exchange, and knowledge to action. A widely used definition of knowledge translation, developed by the Canadian Institutes of Health Research, describes a dynamic and iterative process that incorporates synthesis, dissemination, exchange, and ethically sound application of knowledge to improve health within a complex system of interactions between researchers and knowledge users [8]. Implementation science is a related field involving the study of methods and strategies that facilitate the uptake of research into regular use by policymakers and practitioners to support evidence-informed decision-making [9]. Also relevant is science communication, which is often an important component of the research translation process, particularly in relation to community engagement and public participation [10]. Science communication is a cross-cutting theme for the HEAL network.

The HEAL research translation subgroup acknowledges that successful translation and implementation require a continued shift away from traditional, siloed research and policy activities and ongoing strengthening of relationships between communities, practitioners, researchers, and policy actors [11,12]. Such a shift requires an ongoing cultural change to incentivise, reward, and enact opportunities for collaboration, coordination, and partnership, and overcome the barriers of working across sectors. Existing barriers are well-recognised and include cultural differences between communities, researchers, practitioners, and policy actors; limited existing relationships and opportunities for engagement between relevant parties; institutional factors such as unsupportive leadership or complex organisational structures; and conflicting priorities, timelines, and resource demands. Other challenges include a constrained understanding of existing policy processes among researchers, communities, and practitioners, as well as relevant political and bureaucratic restraints, and likewise, different expectations of co-designed research processes among policy actors [8,9,13,14].

Addressing barriers to research translation requires consideration of the different needs of the community, community organisations, and research, policy, and practice groups; culture within institutions; capacity and interest of involved stakeholders; leadership and governance arrangements; and political, social, and economic influences [14,15]. Establishing trusting relationships is particularly important [15].

Furthermore, it is important that research translation approaches consider the type of knowledge being translated, the reason for translation, and the target audience or user of the knowledge [14]. Critically, the term ‘knowledge’ may not refer only to empirical research but to personal experience or expertise and to collective or institutional knowledge and memory [14]. Recognising the breadth of knowledge types is essential and draws on the diversity of contributors to HEAL and the central role of First Nations Knowledge Systems [16] which is a cross-cutting priority across HEAL. The explicit valuing of First Nations Knowledges, as well as experience and expertise from policy and practice domains, are essential in supporting the co-design, interpretation and translation of research. The embedding of First Nations Knowledge Systems seeks to ensure the systematic engagement of, and leadership by, Aboriginal and Torres Strait Islander Elders, people, and organisations in the work of HEAL. For example, the First Nations Steering Committee provides cultural governance and strategic advice on engagement processes, research impact, and strengthening of research capacity. In addition, it seeks to elevate, strengthen and leverage the inter-generational knowledge developed by Aboriginal and Torres Strait Islander communities over millennia and weave this with other forms of scientific understanding [7,16].

It is also acknowledged that methods to support research translation vary and have evolved over time. Previously, ‘push and pull’ strategies were common, whereby researchers ‘push’ out knowledge to users, for example through research papers, policy briefings, and evidence syntheses, while policy actors ‘pull’ knowledge into their decision-making processes [14]. There is, however, increasing the prominence of integrated approaches which have a stronger emphasis on knowledge exchange and collaboration between stakeholders, including, for example, community members, policy actors, and service providers [14]. The HEAL Network is prioritising integrated approaches to research translation, recognising the value of broader forms of knowledge and the importance of First Nations Knowledge Systems and experience and expertise from outside the traditional research environment. HEAL’s approach focuses on co-design and participatory approaches across the spectrum of priority-setting, research goals, research question development, and research translation and impact to ensure that a wide range of knowledge types inform real-world decision making.

## 4. Other Guiding Frameworks

In addition to research translation theory, the subgroup has utilised a range of relevant existing frameworks to inform HEAL’s approach to translation and implementation. In particular, the principles of Health in All Policies (HiAP) and Environment in All Policies (EiAP) have been drawn upon [17,18].

HiAP recognises that factors influencing health are frequently outside the health sector, hence health considerations are important across all policy domains. The HiAP approach ‘systematically takes into account the health implications of decisions, seeks synergies, and avoids harmful health impacts in order to improve population health and health equity’ [17]. Relevant principles include transparent and open communication, investment in trust and relationships, respectful and responsive approaches to partners’ needs, flexibility and adaptability, and joined-up approaches [19]. HiAP ways of thinking and ways of working are also important, for example valuing partnerships, citizens and the community at the centre, outcome-focused, and enacting co-design, co-production, and collaboration to achieve shared goals and realise co-benefits [19]. HiAP principles are mirrored in other frameworks such as Healthy Cities which was initiated by the World Health Organization in response to urbanisation-related health issues [20]. The creation of healthy cities requires good governance, political commitment, collaboration, transparency, and community participation to ensure that health equity and human development are central to policies and decision-making in urban settings [20].

The concept of EiAP parallels that of HiAP, whereby an environmental lens is systematically applied to policy development across all sectors, considering the environmental impact and consequences, both intended and unintended, of decisions [18]. EiAP recognises that many determinants of environmental health lie outside the domain of environmental sciences and policy, and that addressing such determinants benefits both the environment and broader society [18].

For the HEAL Network, the application of both health and environment in all policy approaches supports cross-sectoral collaboration and fosters inter-disciplinary policy, research, community, and practitioner relationships. Such relationships will support capacity development across HEAL as stakeholders seek to address research and policy needs with common objectives and mutually beneficial outcomes: improvements to human and environmental health, and increased resilience to climate change.

## 5. The HEAL Network’s Research Translation, Implementation, and Impact Strategy

Informed by the aforementioned paradigms and frameworks, the primary task of the HEAL Network’s research translation subgroup was the development of a research translation, implementation, and impact strategy for HEAL. The strategy supports the interwoven objectives of policy and community-relevant research, strengthened relationships between researchers, policy actors, practitioners, and community members, and capacity development across all facets of research translation and implementation.

The strategy is based on key values of the HEAL Network, encouraging the co-design, synthesis, and application of scientific evidence to policy and practice in systematic, timely, measurable, ethical, and sustainable ways that uphold HEAL’s aim and objectives. Across the HEAL Network, it is expected that research translation and implementation are incorporated into co-design processes from the outset (from priority identification onwards) and that research is clearly linked to outcomes and long-term impact. It is also essential that community priorities, values, and needs are central to HEAL’s activities to ensure research is relevant and findings readily utilised to generate real-world impact. The strategy supports the involvement of community members and organisations to define policy problems, identify priorities, shape research questions, and implement findings through Communities of Practice which serve as fora for local knowledge exchange.

### 5.1. Principles

Through an iterative process, the HEAL Network’s research translation subgroup developed a set of key principles to underpin the research translation, implementation, and impact strategy. The principles are outlined in Box 1.

Box 1 **Key Principles: Research translation, implementation, and impact strategy for the HEAL Network.**

■ Research is rigorous, ethical, and underpinned by a shared purpose to achieve the aim and objectives of the HEAL Network
■ Members of the HEAL Network are committed to community-, policy-, and practice-relevant research and to supporting research impact
■ Aboriginal and Torres Strait Islander concepts, culture, and ways of knowing are respectfully considered and integrated into all aspects of the HEAL Network’s activities
■ All stakeholders are valued and empowered to contribute including at-risk groups, people with lived experience, community organisations, researchers, policymakers, practitioners, and the wider community; power imbalances are identified, acknowledged, and addressed
■ Each part of the HEAL Network (for example, each Community of Practice) has a Research Impact Plan to ensure that all activities consider policy and practice needs and opportunities
■ Research is largely action-oriented to facilitate involvement and knowledge sharing among all stakeholders
■ Opportunities for, and commitment to, research translation, implementation, and impact are considered in the distribution of funds across the HEAL Network
■ Responsibility lies with all members of the HEAL Network to generate knowledge that is policy-relevant, is a community priority, and supports meaningful, sustainable outcomes
■ Reciprocal relationships build trust and respect across the research, policy, and practice communities, including the HEAL Network’s Communities of Practice, as well as with the broader community; communication facilitates the productive intersection of needs and interests
■ Collaboration creates dynamic, inclusive, and porous communities of researchers, community members and organisations, policymakers and practitioners; strong partnerships support sustainable outcomes
■ Capacity development is prioritised across the HEAL Network to support skill and knowledge development and promote agile, innovative approaches to research translation and implementation

### 5.2. Actions

Preliminary actions to support research translation and implementation across the HEAL Network are outlined in the strategy and under ongoing co-design, with the research translation subgroup continuing to meet on a regular basis. Proposed additions to the subgroup membership will support broader engagement through increased involvement of community members, practitioners, and policy actors. Importantly, the subgroup will facilitate connections within and beyond the HEAL Network, with a focus on networking, support for multi- and inter-disciplinary collaborations, and strengthening the voices of community-based contributors and policy actors. HEAL members will be supported to identify and action opportunities for research impact and to share their experiences, challenges, and successes. As a translation lens is encouraged across HEAL, all parts of the network are responsible for establishing a research translation, implementation, and impact plan relevant to their scope of work; plans will be prepared with support from the subgroup.

Across the HEAL Network, it is expected that funding and other relevant decisions will prioritise activities that support research translation, implementation, and impact. A program for capacity development opportunities is being established, including, for example, workshops, webinars, and an online translation, implementation, and impact knowledge hub. Specific capacity development opportunities will be offered to early career participants. Also, HEAL members will be encouraged to document and share examples of effective research translation, implementation, and impact, for example through case studies which will also support capacity development.

The experience and needs of HEAL members and affiliates will be explored through an online survey planned for 2023. The survey comprises 39 multiple-choice and free-text questions. It will assess the baseline knowledge, attitudes, and practices of respondents in relation to establishing and maintaining successful research, policy, and practice collaborations. The results of the survey will help identify opportunities for sharing research translation experience and expertise, improved engagement, and capacity development across the HEAL Network. The survey findings will also inform the ongoing work of the research translation subgroup, for example by providing information about barriers to research translation across HEAL and enablers of successful collaboration, which can be used to inform capacity development activities.

### 5.3. Monitoring and Evaluation

A monitoring and evaluation plan for research translation, implementation, and impact across the HEAL Network is under development. A range of potential indicators of successful research translation and implementation have been identified to support monitoring and evaluation, such as knowledge, attitudes, and practices of HEAL members, engagement and strength of relationships between HEAL members, other stakeholders, and the wider community, the range of translation and implementation approaches taken across the HEAL Network, and access of policymakers, practitioners and the wider community to researchers.

The HEAL Network survey described above will support the monitoring and evaluation of research translation knowledge, attitudes, and practices among HEAL members and affiliates. It is expected that the initial survey will provide baseline data, with a follow-up survey planned to track changes over time.

The monitoring and evaluation process will support ongoing review of research translation and implementation across HEAL, including identification of ‘what works’ and opportunities for improvement. The findings will be important when planning for activities beyond HEAL, to ensure the sustainability of successful research translation and implementation approaches across the network.

## 6. Conclusions

The HEAL Network has been established to improve and strengthen health, the Australian health system, and the environment and comprises community members and organisations, policy actors, practitioners, researchers, and other stakeholders from across the country. Research translation, implementation, and impact are key objectives across HEAL and are essential to achieve the network’s intended outcomes. A research translation subgroup comprising members of the HEAL Network has been established and a research translation, implementation, and impact strategy developed. The strategy outlines a range of principles underlying research translation across HEAL along with proposed actions and opportunities for monitoring and evaluation. Actions and indicators remain under development with the subgroup continuing to meet in 2023. Through these actions, the HEAL Network is well-placed to support ongoing, constructive relationships across the research and policy environments and the wider community. Such relationships will support successful research translation and implementation through a culture of collaboration to address critical issues for human and environmental health.

## Data Availability

Not applicable.
